# Intracytoplasmic dot‐like inclusions as cytopathologically useful findings of ependymoma: Case report of adolescent supratentorial anaplastic ependymoma with clear cell morphology

**DOI:** 10.1002/ccr3.3536

**Published:** 2020-11-16

**Authors:** Taku Homma, Reina Mizuno, Yu Miyama, Tomonari Suzuki, Eita Uchida, Jun‐ichi Adachi, Masanori Yasuda

**Affiliations:** ^1^ Department of Pathology Saitama Medical University International Medical Center Saitama Japan; ^2^ Department of Neuro‐Oncology/Neurosurgery Saitama Medical University International Medical Center Saitama Japan

**Keywords:** EMA, ependymoma, intracytoplasmic dot‐like inclusion, L1CAM, RELA fusion, supratentrial

## Abstract

Pediatric supratentorial ependymomas often have a clear cell morphology and reveal a *RELA* fusion. When a clear cell neoplasm is intraoperatively diagnosed, intracytoplasmic dot‐like inclusions by cytology are a useful cytopathological feature of ependymoma.

## Graphical abstract

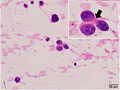

## 1 INTRODUCTION

Ependymomas are major primary glial neoplasms that mainly affect children and young adults, accounting for 2%‐9% of all neuroepithelial tumors.^1^, ^2^ Most ependymomas arise in the infratentorial area, including the fourth ventricle and spinal cord. However, although rare, supratentorial parenchymal ependymomas (ST‐EPNs) can also occur, mainly affecting pediatric patients.^1^, ^2^ Furthermore, pediatric ST‐EPNs often have clear cell morphology, a *C11orf95‐RELA* fusion, and a biologically aggressive course in the clear cell variant.^1^, ^2^, ^3^ Perivascular pseudorosettes are a major pathological hallmark of ependymomas. Additionally, intracytoplasmic dot‐like inclusions are well‐known in ependymoma cell histopathology.^2^ However, these inclusions are not known as an important cytopathological finding for recognizing neoplastic cells as ependymoma cells. Therefore, in this report, we describe the detailed cytopathological and histopathological features of pediatric supratentorial anaplastic ependymoma with clear cell morphology and L1 cell adhesion molecule (L1CAM) immunoreactivity, suggesting *RELA* fusion‐positive ependymoma. We believe that intracytoplasmic dot‐like inclusions in neoplastic cells, in addition to perivascular pseudorosettes, might be a useful finding for ependymomas. Furthermore, we should recognize the presence of *RELA* fusion‐positive ST‐EPNs as a poor prognostic variant of ependymomas.

## 2 CASE REPORT

An 8‐year‐old girl received symptomatic treatment for headache and vomiting in a local pediatric clinic. As her symptoms did not improve, she underwent brain computed tomography (CT) and magnetic resonance imaging (MRI) at a local hospital 6 days after onset. Brain CT and MRI revealed a mass with a cyst at the right temporal lobe. Therefore, subtotal tumor resection was performed 10 days after onset, and the resected brain tumor was diagnosed as anaplastic ependymoma. After the operation, postoperative radiation therapy (59.4 Gy/33 Fr, 1 month) was performed at our hospital, following which the patient was observed at the outpatient ward of the hospital. However, 8 years after the operation, the right temporal tumor recurred and was resected again. As postoperative neuroradiological analysis revealed complete resection of the tumor, she received no postoperative therapy but was followed up at the outpatient ward of the hospital. We observed no tumor recurrence 1 month after the second operation.

## 3 PATHOLOGICAL FINDINGS

### 3.1 Cytopathological findings

**Figure 1:**
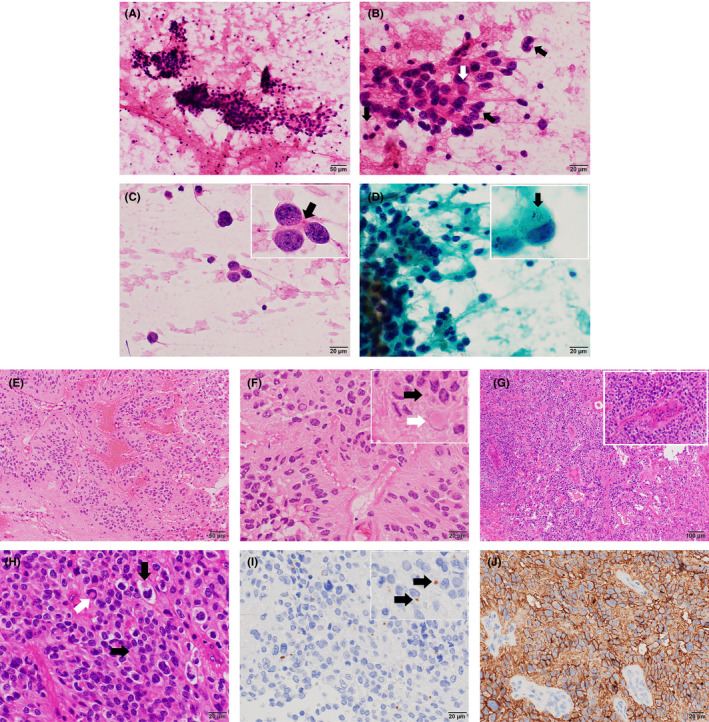
Cytopathological and histopathological features of the present case. A, High degree of cellular clusters without a necrotic background (original magnification: ×200; hematoxylin and eosin [H&E] stain). B, Neoplastic cells with relatively rich eosinophilic cytoplasm, rough fibrillary processes, and round to oval nuclei. On occasion, intranuclear pseudo‐inclusions (white arrow) and/or intracytoplasmic eosinophilic dot‐like inclusions (black arrow) are seen (original magnification: ×600; H&E stain). C, Small nest of neoplastic cells with eosinophilic dot‐like inclusions in their cytoplasm (inset, black arrow) (original magnification: ×600; H&E stain). D, By Papanicolaou staining, intracytoplasmic dot‐like inclusions of neoplastic cells are seen with well‐demarcated shiny oval morphology (inset, black arrow) (original magnification: ×600; Papanicolaou stain). E, Frozen section: Cellular neoplasm with a rich capillary network is observed. Neoplastic cells form perivascular pseudorosette structures (original magnification: ×200; H&E stain). F, Frozen section: neoplastic cells form perivascular pseudorosette. In addition, intranuclear pseudo‐inclusions (inset, white arrow) and intracytoplasmic dot‐like inclusions (inset, black arrow) are detectable in the neoplastic cells (original magnification: ×600; H&E stain). G, Permanent section: High degree of cellular neoplasm with vascular proliferation (inset) (original magnification: ×100; H&E stain). H, Permanent section: neoplastic cells have central, irregular, round, swollen nuclei, and clear cytoplasm, resembling oligodendroglioma, IDH‐mutant and 1p/19q co‐deleted. In part, neoplastic cells have intranuclear pseudo‐inclusions (white arrow) and intracytoplasmic dot‐like inclusions (black arrow) (original magnification: ×600; H&E stain). I, Intracytoplasmic eosinophilic dot‐like inclusions of neoplastic cells positive for epithelial membrane antigen (inset, black arrow) (original magnification: ×600; epithelial membrane antigen [EMA]). J, Neoplastic cells reveal L1 cell adhesion molecule (L1CAM) immunopositivity (original magnification: ×400; L1CAM)

A cytopathological specimen was obtained during the second operation via tumor tissue squash‐and‐imprint and stained using the standard Papanicolaou procedure and hematoxylin and eosin (HE) staining (Figure 1A‐D). Cellular clusters without necrotic background were detected (Figure 1A). Neoplastic cells had relatively rich eosinophilic cytoplasm with rough fibrillary processes and round to oval nuclei (Figure 1B). Some neoplastic cells had intranuclear inclusions (Figure 1B) and/or intracytoplasmic dot‐like inclusions (Figure 1B‐C). The intracytoplasmic dot‐like inclusions appeared as eosinophilic dot‐like inclusions in HE staining (Figure 1B‐C) and with well‐demarcated shiny oval morphology in Papanicolaou staining (Figure 1D).

### 3.2 Histopathological findings

In frozen section diagnosis, the tumor specimen had a high degree of cellular neoplasm with perivascular pseudorosettes formed by neoplastic cells elongating their processes into intermingled vessels (Figure 1E, F). Those neoplastic cells often had intracytoplasmic dot‐like eosinophilic inclusions and intranuclear pseudo‐inclusions (Figure 1F). Therefore, for the frozen section specimen, we diagnosed this as being consistent with recurrent anaplastic ependymoma. In permanent section diagnosis, the tumor specimen had a high degree of cellular neoplasm with microvascular proliferation (Figure 1G). Neoplastic cells had irregular‐shaped swollen round nuclei with perinuclear halos and showed clear cell morphology (Figure 1H). Intracytoplasmic dot‐like eosinophilic inclusions and intranuclear pseudo‐inclusions were sometimes detectable in neoplastic cells (Figure 1H). The neoplasm was immunopositive for glial fibrillary acidic protein (GFAP). Furthermore, neoplastic cells frequently had epithelial membrane antigen (EMA, Figure 1I) and/or podoplanin (D2‐40) immunoreactive intracytoplasmic dot‐like inclusions. According to previous reports describing intracytoplasmic dot‐like eosinophilic inclusions of ependymomas,^4^, ^5^, ^6^, ^7^ we considered EMA‐ and/or podoplanin‐positive dot‐like inclusions as intracytoplasmic dot‐like eosinophilic inclusions in the hematoxylin‐eosin‐stained sections. Nuclear expression of alpha‐thalassemia/mental retardation syndrome X‐linked (ATRX) protein was observed, and no mutant isocitrate dehydrogenase 1 (IDH1)‐R132H expression was observed. Neoplastic cells showed diffuse L1CAM expression (Figure 1J). MIB‐1 labeling index of the neoplasm was 7% at the hot spots.

Among those pathological findings, the final diagnosis of the recurrent brain tumor was supratentorial anaplastic ependymoma with clear cell morphology, expressing L1CAM. RNA sequencing from the tumor tissue revealed a fusion of *C11orf95* exon 3 and *RELA* exon 2, the most commonly identified fusion pattern. Therefore, integrated diagnosis of this pediatric supratentorial neoplasm was determined as “ependymoma, *RELA* fusion‐positive” according to the revised 2016 World Health Organization (WHO) classification of central nervous system (CNS) tumors.^8^

## 4 DISCUSSION

Recently, genetic alterations of ST‐EPNs have been reported.^3^ ST‐EPNs with a *C11orf95‐RELA* fusion, in particular, are defined as distinct variants of ependymoma, named “ependymoma, *RELA* fusion‐positive,” in the revised 2016 WHO classification of CNS tumors.^8^ Histopathologically, *RELA* fusion‐positive ependymomas usually have anaplasia, including high cellularity, nuclear atypia, high mitotic activity, microvascular proliferation, and/or tumor necrosis.^3^ Furthermore, they reveal features of clear cell ependymoma (CC‐EPN), classic ependymoma, or papillary ependymoma.^3^ Immunohistochemically, L1CAM expression is known as a surrogate marker of *RELA* fusion in ST‐EPNs.^3^, ^8^ This ependymoma variant is clinically recognized as showing aggressive behavior and a poor prognosis.^8^ In fact, the *RELA* fusion‐positive ST‐EPN affecting this 16‐year‐old girl recurred 8 years after the first operation even though postoperative radiation therapy had been administered. In addition, its histopathology was anaplastic ependymoma with clear cell morphology, resembling CC‐EPN. Furthermore, the neoplasm revealed L1CAM immunoreactivity. These clinicopathological and immunohistochemical features are compatible with a diagnosis of “ependymoma, *RELA* fusion‐positive.” ^3^, ^8^

The ependymoma, in this case, was morphologically characterized by clear cell features. CC‐EPN is a rare histological variant of ependymoma, accounting for 6% of ependymomas.^1^ Most CC‐EPNs affect the supratentorial region of young patients.^1^ Histopathologically, clear cell morphology with perivascular pseudorosettes and/or ependymal rosettes is the characteristic feature of CC‐EPNs.^1^ Genetically, supratentorial CC‐EPNs often have *RELA* fusion,^3^ suggesting the association between supratentorial CC‐EPNs and *RELA* fusion‐positive ependymoma.^3^, ^8^, ^9^ Therefore, when diagnosing supratentorial clear cell neoplasms, we have to consider the following differential diagnoses: CC‐EPN, oligodendroglioma, IDH‐mutant and 1p/19q co‐deleted (OD), central neurocytoma (CN), and metastatic clear cell renal cell carcinoma (ccRCC).

The major points of the differential diagnoses of CC‐EPN, OD, CN, and ccRCC are summarized in Table 1.^1^, ^20^ First, clinically, CC‐EPNs affect young patients whereas the other tumors tend to occur in middle‐aged patients.^1^, ^11^, ^12^ The foramen of Monro is the area most frequently affected by CNs, which differs from CC‐EPNs, OD, or metastatic ccRCC. Cytopathologically, neoplastic cells of CC‐EPNs show a cohesive tendency and often form cell clusters suggesting perivascular aggregation.^2^ Furthermore, by detailed cytopathological observation of CC‐EPNs, we found that some neoplastic cells had intracytoplasmic dot‐like inclusions, as has been seen by histopathology.^2^ Histopathologically, intracytoplasmic dot‐like eosinophilic inclusions showing EMA immunoreactivity are reported as helpful diagnostic findings of ependymoma.^6^ These intracytoplasmic dot‐like eosinophilic inclusions of ependymoma were confirmed by electron microscopy as microscopic intracytoplasmic lumina.^6^ Furthermore, these intracytoplasmic dot‐like eosinophilic inclusions could be found in various degrees among various ependymoma subtypes, including ordinary (65%), papillary (33%), clear cell (80%), tanycytic (50%), myxopapillary (20%), and anaplastic ependymomas (66%).^6^ However, to our knowledge, cytopathological descriptions of intracytoplasmic eosinophilic inclusions of ependymoma are rare.^21^ Otani et al reported two cases of ependymoma with intracytoplasmic inclusions: One was an ordinary WHO grade Ⅱ ependymoma with perivascular pseudorosettes, and the other was ependymoma characterized by signet‐ring‐like neoplastic cells, and they suggested that the presence of intracytoplasmic inclusions in cytological specimens of brain tumors could be useful for the diagnosis of ependymomas.^21^ Although there are very limited cases, we also suggest that the presence of intracytoplasmic dot‐like inclusions in cytology might be a useful cytological finding suggesting ependymal neoplasm even when no distinct perivascular pseudorosettes and/or ependymal rosettes are found in a brain tumor. Further case accumulation of cytological specimens of ependymomas and detailed cytopathological analyses of these intracytoplasmic dot‐like eosinophilic inclusions of ependymomas are needed to consider them as cytologically useful findings of ependymoma.

**Table 1:** Summary of clinicopathological features to distinguish between clear cell ependymoma, oligodendroglioma, central neurocytoma, and clear cell renal cell carcinoma

	Clear cell ependymoma	Oligodendroglioma, IDH‐mutant and 1p/19q co‐deleted	Central neurocytoma	Clear cell renal cell carcinoma
Patient age (mean)	Children	Middle‐aged adult	Young adult	Middle‐aged adult
Tumor location	Supratentorial	Frontal lobe	Foramen of Monro	Various areas
Cytomorphological features				
Cell morphology	cohesive monomorphic round cells with fibrillary processes	discohesive monomorphic round cells with scant cytoplasm	discohesive monotonous round cells with scant cytoplasm	cohesive cells with granular vacuolated cytoplasm
Nuclei	round	round and uniform	round and uniform	round
Nucleoli	inconspicuous	inconspicuous	inconspicuous	conspicuous
Inclusion	intracytoplasmic dot inclusion	none	none	none
Other features	perivascular aggregation	delicate curved vessels	acellular fibrillary clump	none
Histopathological features				
Growth pattern	sheet pattern with perivascular pseudorosette	sheet pattern with chicken‐wire capillary network	sheet with acellular neuropil‐like area	glandular, alveolar, and/or tubular pattern with rich capillary network
Cell morphology	round to oval neoplastic cells with clear cytoplasm	neoplastic cells with round nuclei and perinuclear halo	neoplastic cells with round nuclei and perinuclear halo	polygonal cells with clear cytoplasm
Necrosis	varies between cases	−	−	varies between cases
Immunohistochemical features				
GFAP	+	+ (minigemistocyte, gliofibrillary oligodendroglia)	−	−
EMA	+ (dot/ring)	−	−	+ (membranous)
AE1/AE3	+ (positive sometimes)	−	−	+
Synaptophysin	−	+ (positive sometimes)	+	−
CD10	−	−	−	+
L1CAM	+	−	−	−
IDH1 R132H	−	+	−	−
Genetics				
*C11orf95‐RELA* fusion gene	present	−	−	−
1p/19q co‐deletion	−	present	−	−

Immunohistochemically, CC‐EPN shows GFAP immunoreactivity^2^ and EMA‐immunopositive intracytoplasmic dot‐like inclusions.^3^ Furthermore, as seen in our case, supratentorial CC‐EPN tends to express L1CAM, suggesting the presence of *C11orf95‐RELA* fusion.^3^ These immunohistochemical features are also useful for excluding OD, CN, and ccRCC.

In conclusion, there are several histologic types of brain tumors showing clear cell morphology. Among them, some show biologically aggressive behavior and require suitable postoperative chemoradiation therapies. One of those tumors is ST‐EPN with *RELA* fusion. It is difficult to diagnose a *RELA* fusion‐positive ependymoma by intraoperative‐frozen pathological diagnosis. However, using the patient's age, tumor location, and several cytopathological findings including the intracytoplasmic dot‐like inclusions of neoplastic cells, we could suggest a diagnosis of ependymoma with a possible *C11orf95‐RELA* fusion: the poor prognostic variant of ependymoma.

## CONFLICT OF INTEREST

None declared.

## AUTHOR CONTRIBUTIONS

Conceptualization: Taku Homma. Patient's care: Reina Mizuno, Tomonari Suzuki, Eita Uchida, Jun‐ichi Adachi. Pathological investigation: Taku Homma, Yu Miyama, Masanori Yasuda. Writing – original draft: Taku Homma. Writing – review and editing: Taku Homma.

## ETHICAL STATEMENT

Appropriate consent has been obtained.

## Data Availability

The data that support the findings of this study are available from the corresponding author upon reasonable request.
